# Role of granulocyte transfusions in combating life-threatening infections in patients with severe neutropenia: Experience from a tertiary care centre in North India

**DOI:** 10.1371/journal.pone.0209832

**Published:** 2018-12-27

**Authors:** Akanksha Garg, Anshul Gupta, Ashish Mishra, Manoj Singh, Sanjeev Yadav, Soniya Nityanand

**Affiliations:** Department of Hematology, Sanjay Gandhi Post Graduate Institute of Medical Sciences, Raebareli Road, Lucknow, India; University of Tsukuba, JAPAN

## Abstract

Bacterial and fungal infections still remain an important cause of mortality in patients with hematological malignancies and in recipients of hematopoietic stem cell transplants (HSCT) especially in developing countries like India. Granulocyte transfusions (GTX) from healthy donors may lead to early clearance of index infection and thus prevent mortality. The aim of the present study was to evaluate the efficacy of GTX in combating life-threatening infections and preventing mortality in patients of hematological disorders/recipients of HSCT with severe neutropenia. This study was a prospective, observational analysis of patients with different hematological disorders/recipients of HSCT, who received GTX from January 2014 to December 2017. All patients had an Absolute neutrophil Count (ANC) < 0.5 x 10^9^/L and a life threatening sepsis defined by presence of hemodynamic instability/ impending septic shock/ continuous high fever despite the use of the highest line of antimicrobials. A total of 143 granulocyte collections were done for 66 infectious episodes (IEs) in 60 patients. Multidrug resistant organisms (MDROs) were observed in 47/66 IEs (71.2%) and fungal infections were seen in 9/66 IEs (13.6%). Resolution of index infection after GTX was seen in 45/66 IEs (68.2%), and the 30 day overall survival (OS) was 67.7%. OS was significantly higher in patients who received GTX within 7 days of neutropenic sepsis (p = 0.01). Patients with MDROs who received early GTX therapy had a better OS as compared to those who received late GTX (p = 0.02). GTX were well tolerated and only 6 patients’ developed mild features of transfusion related acute lung injury (TRALI) which was managed conservatively, and 1 patient demonstrated hypocalcemic tetany. GTX may be of particular relevance in countries like India, where the incidence of infections is very high in neutropenic patients and there is an increasing emergence of MDROs.

## Introduction

Despite modern antimicrobials and supportive therapy, bacterial and fungal infections are still major complications in patients of hematological malignancies and in recipients of hematopoietic stem cell transplants (HSCT) with treatment-related neutropenia. Thirteen to 60% of HSCT recipients develop blood stream infections (BSI), which are associated with 12% to 42% mortality **[1].** In recent years, the Infectious Diseases Society of America (IDSA) has highlighted a fraction of antibiotic-resistant bacteria (*Enterococcus faecium*, *Staphylococcus aureus*, *Klebsiella pneumoniae*, *Acinetobacter baumannii*, *Pseudomonas aeruginosa* and *Enterobacter*spp.)–Acronymically named ‘the ESKAPE pathogens’–capable of ‘escaping’ the biocidal action of antibiotics. These multi- drug resistant organisms (MDROs) are responsible for a significant number of nosocomial infections **[2].** MDROs are defined as microorganisms, predominantly bacteria that are resistant to one or more classes of antimicrobial agents **[3].** In the last decade there has been an increase in the prevalence of MDRO world over, which is much more alarming in India as compared to Western populations **[4, 5].** In a surveillance study in pediatric oncology patients from a cancer center in India in Mumbai, a prevalence of community acquired MDRO colonization especially due to carbapenem resistant Enterobacteriaceae was as high as 60% and was associated with a mortality of 58% to 70% **[6].** Needless to say, mortality due to these agents during neutropenia is extremely high and negates the potential benefits of intensive chemotherapy regimens. Antimicrobials often fail to combat MDROs and there is a need for alternate therapeutic approaches.

Functioning white blood cells are a vital component of the natural defence system against infection in humans. Since patients with haematological malignancies have either neutropenia or neutrophil dysfunction, granulocyte transfusions (GTX) from healthy donors providing extrinsic neutrophils may lead to early clearance of index infection hence promoting survival of patients when antimicrobials fail to combat the infection. Different studies have shown varying efficacy of GTX and there is paucity of data from India where GTX may have a particularly relevant role because of the high prevalence of MDROs. Granulocytes have to be obtained from only blood group matched voluntary donors and thus GTX is an easily feasible option in India, especially if a panel of potential donors have been counselled and screened prior to giving high dose chemotherapy to the patient. We routinely follow this practice in our centre and have rarely faced problem in getting donors in emergency for a neutropenic patient. The aim of the present study was to assess the efficacy as well as the safety of GTX in patients with haematological malignancies/recipients of HSCT facing life-threatening infections, not responding to highest line of antimicrobial therapy.

## Materials and methods

### Patient criteria

We conducted a prospective observational analysis of all patients who received GTX for severe neutropenic sepsis while undergoing treatment for any haematological condition in the Dept of Hematology, during the period January 2014 to December 2017. One hundred and forty three granulocyte collections were done for 66 infectious episodes (IE) in 60 patients.

Patient records were anonymized and de-identified prior the analysis. The Research Committee of Department of Hematology and the Institutional Ethics Committee of Sanjay Gandhi Post Graduate Institute of Medical Sciences approved the study.

The objective was to determine the efficacy and safety of GTX in patients of all age groups with haematological disorders having an absolute neutrophil count (ANC) < 0.5 x 10^9^ /L with life threatening sepsis (presence of hemodynamic instability/ impending septic shock/ hectic fever), not responsive to highest line of antimicrobials (including antifungals). The antimicrobial policy was uniform amongst all patients. With the onset of fever, after obtaining blood culture specimens from central line and/peripheral line, the first line empirical antibiotic of choice was Piperacillin-Tazobactum ± Amikacin. If there was a suspicion of soft tissue infection and/or the fever persisted over the next 48 hours Teicoplanin was added. Besides sending blood cultures, cultures of specimens from other clinically probable sites of infection (e.g sputum, urine, perianal, synovial fluid etc) were also sent, and the culture reports were aggressively followed up and once available the antimicrobial drugs were accordingly modified. However, in patients who presented/developed features of severe sepsis along with hemodynamic instability, the antibiotics were quickly upgraded to Carbapenem (preferably Inj. Meropenem) followed by addition of Inj. Colistimethate as the incidence of multidrug resistant gram negative infections in our hospital is high (Incidence of Extended spectrum β lactamase producing Gram negative bacteria is 70–80%, carbapenemase producing Pseuodomonas is 40–45%, carbapenemase producing Klebsella is 50–55% and carbapenemase producing other Enterobacteria is 20–30%,) (data from the Microbiology Dept). A fungal infection was suspected if fever persisted for more than 96 hours despite upgrading the antibiotics with no evidence of any bacterial growth in the cultures and an empirical antifungal drug (usually Inj. Amphotericin B) was added. High resolution CT Chest/Sinuses (if clinically indicated) was done in these patients to look for any radiological evidence of active mold fungal infection (usually invasive Aspergillosis) so that the antifungal drug could be modified accordingly. Once the counts started recovering, GTX therapy was stopped and once the patients were hemodynamically stable and afebrile, the antibiotics were withheld.

### Primary Outcome measures

- Resolution of the IE.
- Survival at 30 days.

### Secondary outcome measures

- Frequency and nature of adverse reactions to GTX.

### Donor mobilisation and granulocyte collection

One-hundred and forty three granulocyte apheresis were done in 138 healthy voluntary donors. Prerequisites for donation were an informed consent, ABO and Rhesus blood group compatibility, appropriate CMV status, normal complete blood counts and negative serology for viral hepatitis and HIV. Granulocyte donors were mobilised with 10 μg/kg G-CSF subcutaneously and 8 mg of dexamethasone administered intravenously 12 h before each granulocyte collection. Apheresis was performed using a Spectra Optia system, using 30ml of trisodium citrate (46.7%) suspended in 500ml of 6% hydroxyethyl starch as anticoagulant. Target granulocyte collection was a minimum of 50 x 10^9^ granulocytes/ product. The final volume of the product was decided on the basis of granulocyte count achieved at the end of the first 100 ml of granulocyte collection. Before transfusion, the apheresis product was irradiated with 25Gy.

### Granulocyte transfusions

Granulocytes were transfused within 6 hours of collection, over a period of 1–2 hours in adults and 2–3 hours in pediatric patients, with monitoring of vital parameters. Premedication with 15mg/kg of acetaminophen, 4mg/kg of hydrocortisone and 0.1 mg/kg of chlorpheniramine maleate was given prior to each transfusion. Each patient was given 2 ml/Kg (maximum of 10ml) of calcium gluconate (10%) before, during and at the end of transfusion to avoid hypocalcemic tetany.

### Statistical evaluation

Chi square test or Fisher’s exact test was used for categorical variables and Mann Whitney U test for continuous variables. A significance level of 0.05 was used. SPSS version 20.0 was used (SPSS Inc., Chicago, IL, USA). Interquartile ranges (IQR) were used for categorical variables. IQR is the difference between 75th and 25th percentiles, or between upper and lower quartiles, i.e., Q_3_ − Q_1_. Survival was compared between treatments using Kaplan-Meier curves and log-rank test statistics.

## Results

### Characteristics of the donor and the granulocyte product

Of the 138 donors, 129 were males and 9 females and the median age was 30 years (IQR: 24–38 years). The median Total Leukocyte Count (TLC) of the donors 12 hrs after the G-CSF and dexamethasone injections was 33.5 x10^9^/L (IQR: 29.5–39.5x10^9^/L). The median granulocyte dose for an IE was 57.7x10^9^ granulocytes/ product/ IE (IQR: 47–68.4x10^9^ granulocytes/ product/ IE), the median granulocyte dose/kg/IE was 10.4x10^8^ granulocytes (IQR: 8.8–14.4x10^8^ granulocytes/kg/IE) and the median volume of collection was 550ml (IQR: 500–600 ml). The apheresis procedure was well tolerated, and only 6 donors reported the use of an analgesic drug for mild bone pains.

### Patient characteristics

Sixty patients were given GTX for 66 IE, 41 were males and 19 were females. The median age of patients was 21 years (IQR: 16–45 years) and 21 patients were less than 18 years of age. The diagnoses of the patients were as follows: acute myeloid leukemia (n = 24), acute lymphoblastic leukemia (n = 9), severe aplastic anemia (n = 15), mixed phenotypic acute leukemia (n = 1), non Hodgkin lymphoma (n = 3), therapy related AML (n = 1), multiple myeloma undergoing autologous stem cell transplant (n = 3), myelodysplastic syndrome (n = 1), allogeneic HSCT recipients (n = 3; all for severe aplastic anemia). The median duration of fever before giving GTX was 7 days (IQR: 5–12 days). The median number of GTX given per patient was 2 (IQR: 1–3). The median baseline TLC of the patients was 0.3 x10^9^/L (IQR: 0.1–0.7 x10^9^/L), and the median TLC 6 hours after administration of the product was 1.5x x10^9^/L (IQR: 0.8–2.8 x10^9^/L) with a median increment from baseline of 1.2 x10^9^ /L (IQR: 0.3–2.5 x10^9^/L). The median duration of severe neutropenia was 15 days (IQR: 10–23 days).

### Microbiological profile of patients

Out of the total 66 IEs, bacterial blood cultures were sterile in 18 IEs whereas 48 IEs had positive bacterial cultures. Growth of *methicillin sensitive staphylococcus aureus* (MSSA) in sputum occurred in one IE whereas in the all the other IEs, MDROs were isolated. More than one MDRO was isolated in some of the IEs. The details of the MDROs isolated are given in Table 1. Of these 18 patients, fungal infections were seen in 9/66 IEs. Culture proven fungal infections were seen in 4 patients (1 patient had candida isolated from blood and 3 patients had aspergillus isolated from sputum), and 5 patients had a clinically probable fungal infection since CT chest was suggestive of invasive aspergillosis. The rest 9 patients with culture-negative sepsis demonstrated high pro-calcitonin values, and all of these patients had evidence of infection (4 had pneumonia, 4 had GI sepsis and 1 had necrotising fasciitis).

**Table 1:** Multi drug resistant organisms (MDROs) during the infectious episodes (IEs).

ORGANISM	No. of IE	SITE(n)
Klebsiella	23	Blood(10), sputum(6), groin pus(1), perianal(1), urine(3), axillary ulcer(1), synovial fluid(1)
Pseudomonas	2	Blood(2)
Methicillin Resistant Coagulase Negative Staphylococcus (MRCONS)	7	Blood(1), sputum(5), perianal(1)
Acinetobacter	4	Sputum(2), urine(1), perianal(1)
Providentia	1	Perianal(1)
Methicillin Resistant Staphylococcus Aureus	3	Sputum(3)
Serratia	1	Blood(1)
Enterococcus faecium	2	Urine(1), perianal(1)
Citobacter koseri	2	Blood(1), axillary ulcer(1)
Escherichia coli	4	Blood(3), cvp line tip(1)
Stenotrophomonas maltophilia	2	Blood(1), tracheal aspirate (1)
Morganella	1	Urine(1)
Enterobacter	1	Blood(1)
Sterptococcus	2	Blood(1), sputum(1)

### Primary outcome

**Fig 1:**
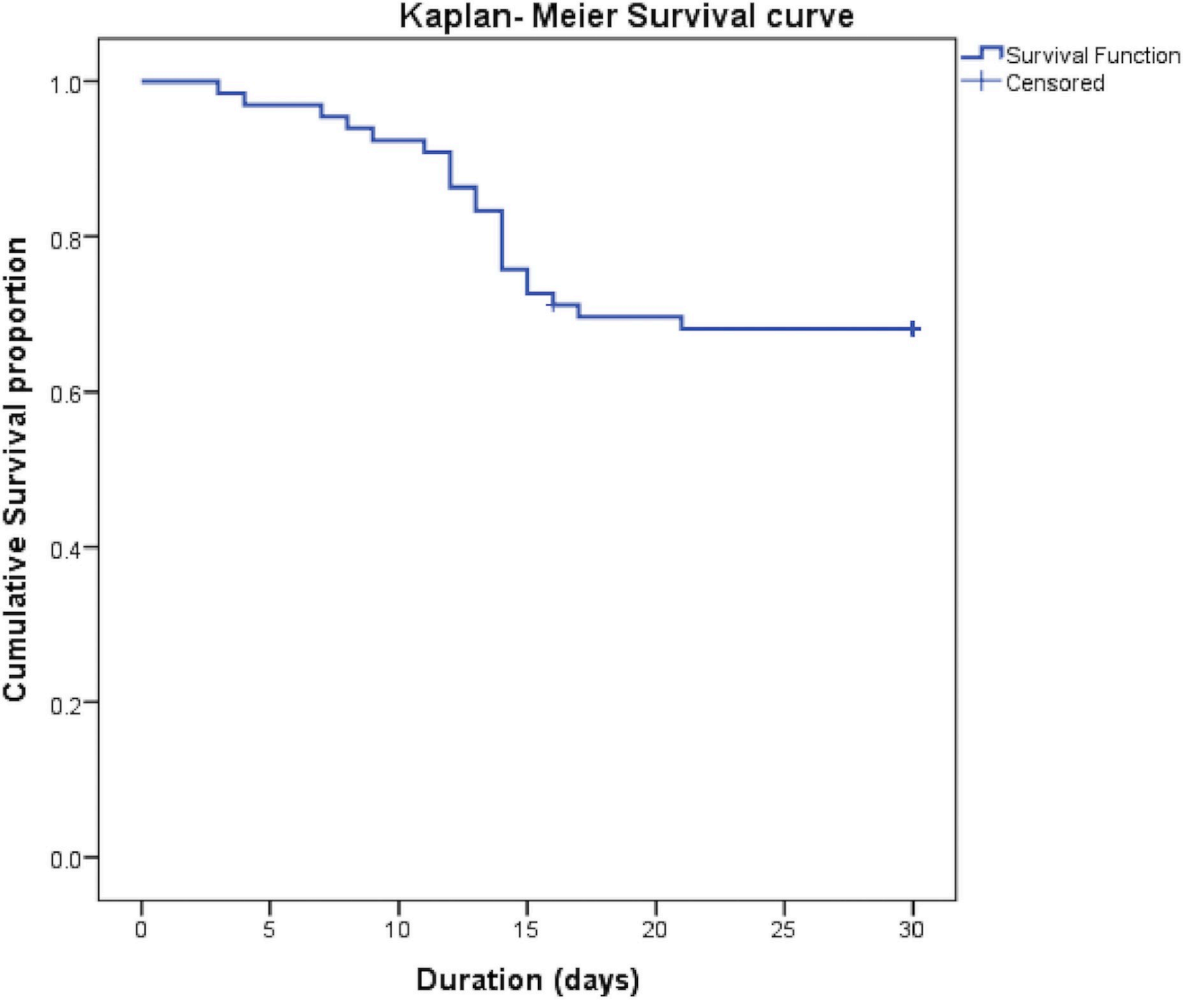
Kaplan Meier survival curve at 30 days of patients given granulocyte transfusions (GTX).

Resolution of the index infection was seen in 45/66 IEs (68.2%). The 30-day survival was 67.7% (Fig 1).

**Fig 2:**
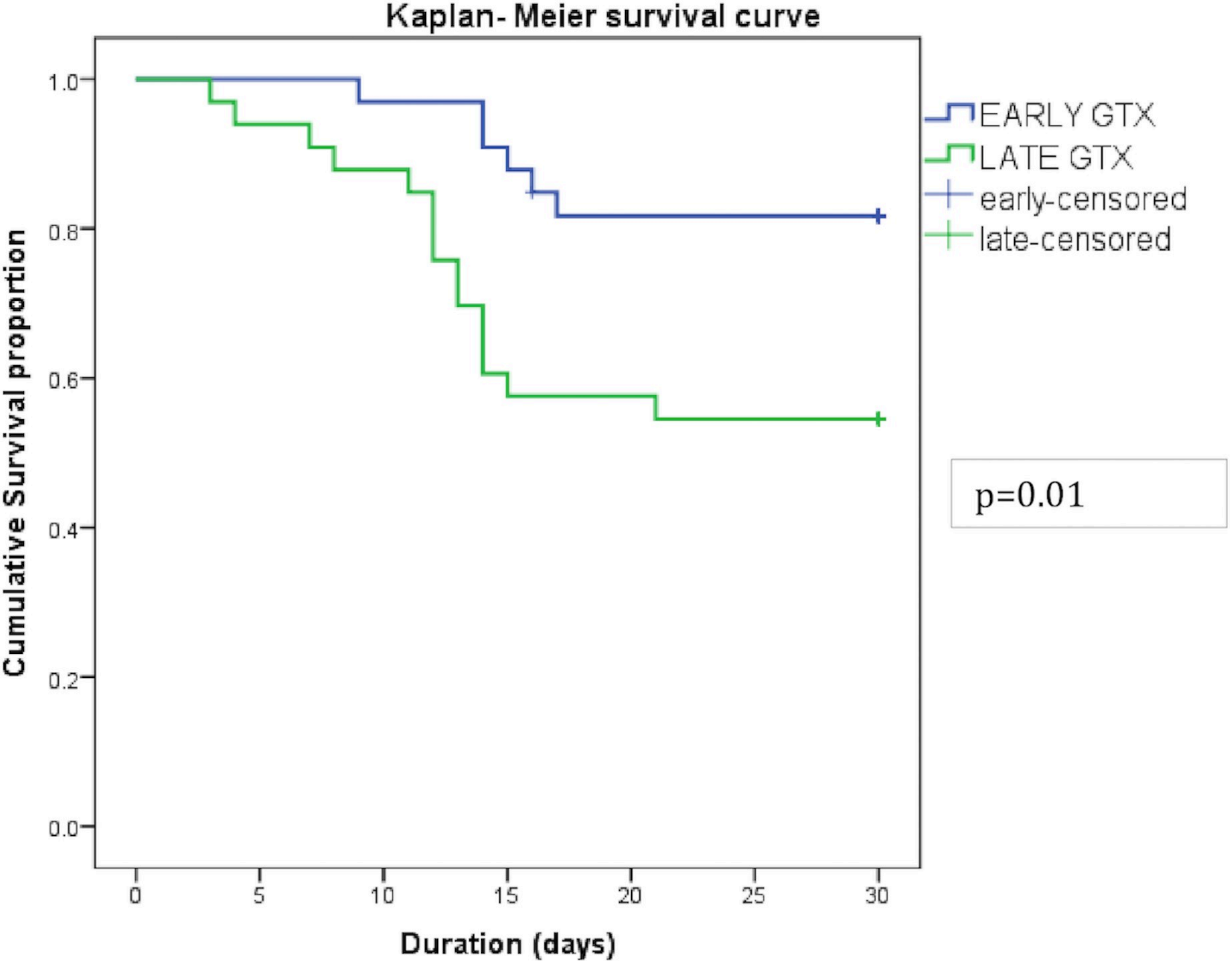
Kaplan Meier survival curves for infectious episodes (IEs) given GTX before 7 days versus those given GTX ≥7 days.

Thirty day survival was better in those IEs where GTX therapy was instituted early i.e,within 7 days of neutropenic sepsis in comparison to those who received GTX after 7 days (Log-rank test p value = 0.01) (Fig 2). Durations of fever and severe neutropenia were shorter in patients who received early GTX within 7 days of the IE (8 days vs 19 days; p = 0.001 and 17 days vs 28 days, p = 0.001, respectively) (Table 2).

**Table 2:** Early versus Late GTX.

PARAMETER	Early GTX(Mean±SD) (<7 days) (n = 33)	Late GTX(Mean±SD) (≥ 7 days) (n = 33)	P value
Median GTX Dose /kg/IE (IQR) (x10^8^granulocytes/kg/IE)	10.5 (9.3–14.4)	10.1 (8.1–13.2)	0.851
Total days of fever	8.9± 6.3	19.4 ± 7.7	***0*.*001***
Days for fever defervescence	2.2± 3.0	3.1± 2.6	0.18
Days of severe neutropenia	16.7± 21.9	27.9± 28.1	***0*.*001***
Baseline TLC (x 10^9^/L)	0.41±0.43	0.41 **+**0.6	0.34
6 hour TLC (x 10^9^/L)	1.51±1.22	2.64 ±2.24	0.53
Increment in TLC (x 10^9^/L)	1.23±1.13	2.04 ±1.95	0.11

**Fig 3:**
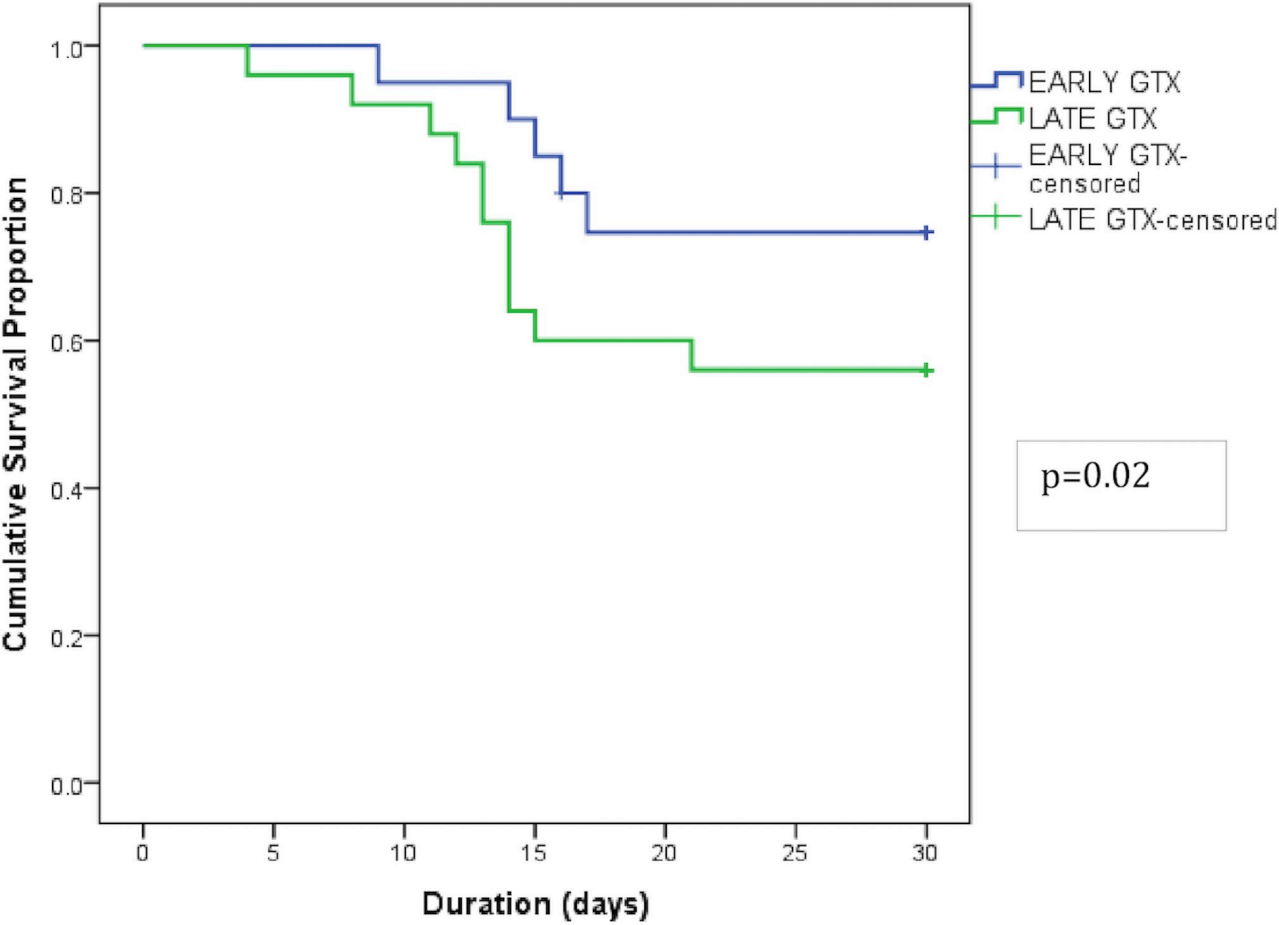
Kaplan Meier survival curves for infectious episodes (IEs) due to MDROs given GTX before 7 days (n = 20) versus those given GTX ≥7 days (n = 27).

Of the 47 IEs due to MDROs, patients in 36 IEs (76.6%) could be successfully salvaged and only patients in 11 IEs (23.4%) succumbed. In these 47 IEs due to MDROs, in 20 the GTX were administered early (<7 days) and in 27, the GTX were given late (≥7 days). GTX therapy when instituted early, was associated with a better survival (Log- rank test p value = 0.02) (Fig 3). With regards to fungal infections seen in 9 cases, 4 patients could be salvaged with GTX and 5 succumbed.

**Fig 4:**
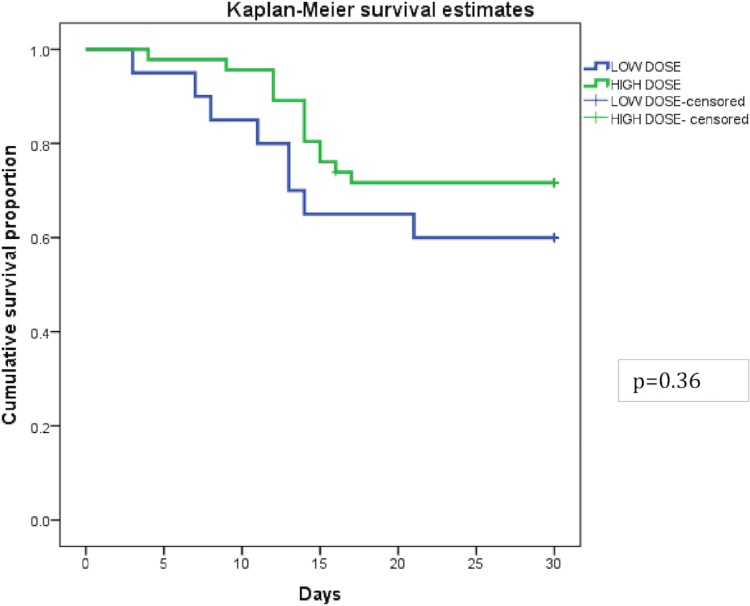
Kaplan Meier survival curves for infectious episodes (IEs) given high dose GTX (≥50 x 10^9^/L) versus low dose GTX (<50 x 10^9^/ L).

There was no significant difference in the 30-day survival between those who received a GTX dose of ≥ 50 X 10^9^ granulocytes/ product/ IE and those who received dose <50 x 10^9^ / product/ IE (Log-rank test p value = 0.36) (Fig 4), though patients who received a GTX dose of ≥50 x 10^9^ / product/ IE (n = 40), showed a higher 6 hour increment in TLC and ANC values in comparison to those who received a GTX dose <50 x 10^9^/ product/ IE (n = 26) (2.53 x 10^9^/L vs 1.1 x10^9^/L, p = 0.01; 2.02 x 10^9^/L vs 0.84 x 10^9^/L, p = 0.02, respectively) (Table 3).

**Table 3:** Low dose versus High dose GTX.

PARAMETER	Low dose(Mean±SD (<50 x 10^9^/product/IE) (n = 26)	High dose (Mean±SD) (≥50 x 10^9^/product/IE) (n = 40)	P value
Total days of fever	19.9 ± 10.3	12.4±7.6	0.85
Days for fever defervescence	2.6 ±2.3	2.5 ±3.1	0.18
Days of severe neutropenia	25.9 ± 26.1	20.6 ± 25.5	0.78
Baseline TLC (x 10^9^/L)	0.35 **±** 0.3	0.43 **±** 0.59	0.35
6hour TLC (x 10^9^/L)	1.1 ± 1.02	2.53 ± 2.04	***0*.*01***
Increment in TLC (x 10^9^/L)	0.84 ± 0.85	2.01 ± 1.79	***0*.*02***

### Secondary Outcome

Six patients developed a mild Transfusion Related Acute Lung Injury (TRALI), which could be managed conservatively, without requiring ventilator support. One patient developed hypocalcemic tetany. Except for mild bony pains in 6 donors, requiring an analgesic drug, no other significant complications were observed in the donors during and after the granulocyte collection.

## Discussion

The efficacy of GTX in resolution of index infection has been demonstrated to be 36.7% to 92.6% in various studies **[7–23]**. The mortality or comparable outcomes such as day-28 or day-30 survival, which is reflective of the mortality related to the infection has been reported to be between 6.7 and 66.7% **[24–35]**. There is one previous study from India in pediatric patients in which resolution of index infection was observed in 54% cases **[36].** There may be several factors responsible for the wide differences reported in these studies including the dose of GTX administered, the time period in the course of infection when administered, patient populations, infection types and a low rate of patient enrolment by participating centres. We observed a resolution of the index infection in 68.2% of our patients, which is higher than reported in several large series. Our 30-day survival was 67.7%, which was comparable to the data of a previous randomized control trial (RCT) in which a survival of 72% was shown **[33]** but higher than most previously published studies.

One reason for the higher efficacy of GTX observed by us could be related to the dose of GTX administered by us. Though it is not very clear which GTX dose predicts their efficacy nor how it should be calculated **[37]** but a number of studies have suggested dose dependency of GTX. Average doses widely differ among the studies from 2.0–15.5x10^8^cells/kg. These differences reflect both the type of recipients, whether adults or children, as well as the type of donors and mobilization protocols **[37]**. Several previous studies have suggested an increased infection control in patients receiving >10x10^9^ granulocytes **[37].** The recently concluded RING study has shown that patients treated with higher numbers of granulocytes per kg body weight (defined as >0.06 × 10^10^ cells/kg) had a significantly better survival at 42 days than did patients without transfusion or with less than 0.06× 10^10^ transfused cells/kg **[38].** In another study it has been observed that patients receiving median doses of 1.5–3.0x 10^8^ granulocytes/kg had reduced infection related mortality. They failed to demonstrate any dose dependent GTX effect in patients with disseminated fungal infections that probably require higher GTX doses **[30]**. In neonates also it has been demonstrated that transfusions of >0.05× 10^10^/kg significantly improved the relative odds of survival **[39].** The median granulocyte dose administered by us for an IE was 57.7x10^9^ granulocytes/ product/ IE (IQR: 47–68.4 x10^9^ granulocytes/ product/ IE) and the median granulocyte dose/kg/IE was 10.4x10^8^ granulocytes/ kg/ IE (IQR: 8.8–14.4 x10^8^ granulocytes/ kg/ IE). We further demonstrated that doses of GTX **≥**50x 10^9^ / product/ IE (equivalent to approximately ≥10x 10^8^ per kg/ IE), though were associated with a higher 6 hour TLC increment (p = 0.02), but did not impact survival at 30 days (p = 0.36). A possible explanation for this could be that there is a plateau of the efficacy of GTX at this dose. Another reason for the higher efficacy observed by us could be that we repeatedly administered GTX every 48 hours till there was a resolution of febrile episode and recovery of life threatening factors and the median numbers of GTX given by us were 3 per IE. Several previous investigators have also repeatedly administered GTX, at 24 hours **[18, 38]**, or every 48 hours **[30]**. In fact, one of the important factors for the efficacy of GTX may be the number of GTX given in succession, which has been brought out by Seidel *et al*, who reported that daily GTX given for at least 5 days and containing a minimum of 3× 108/kg neutrophils per concentrate, was able to generate a stable ANC increment, shorten the duration of neutropenia and support the control of infections in neutropenic patients with high-risk infections **[33].**

The timing of GTX also has a bearing on the resolution of infection and mortality. In a previous study where a very high response rate of 92.6% with GTX was shown, the GTX were instituted early, i.e., after a median infection period of 6 days (range 3–18 days) **[21]**. We have demonstrated that administration of GTX within 7 days of the IE, led to a shorter neutropenic period and a better 30-day survival in comparison to the IEs where given ≥7 days (p = 0.01). Uppuluri *et al*. after changing their protocol from the year 2014 onwards and administering GTX within 48 hours, showed a significant improvement in the survival from 41% to 54% (p = 0.0347) **[36]**. The median GTX dose/kg/IE administered by us was comparable between those who received GTX < 7 days versus those who received GTX ≥ 7 days (p = 0.851), highlighting that the benefit of early administration of GTX was not related to the GTX dose given.

Another noteworthy observation by us was that in IEs due to MDROs, early administration of GTX within 7 days, had a better 30 day overall survival (p = 0.02). This has not been demonstrated in previous studies and highlights that patients with MDROs would benefit with early institution of GTX therapy. Neutropenic infections due to MDROs have a high morbidity and mortality and in countries like India where there is a frequent misuse of over the counter antibiotics and lack of antibiotic stewardship, there has been an alarming increase in the incidence of MDR bacteria in the community and hospitals. In acute leukemia patients, approximately 70% of all blood stream infections have been shown to be due to Gram-negative bacteria and 30–40% of Gram-negative bacterial isolates are resistant to carbapenems **[5, 37]**. In a surveillance study conducted in one of the major cancer centers in South India, it has been observed that in pediatric acute leukemia patients at the time of presentation, 50% of the bacterial isolates from the stool were multi-drug resistant **[40].** We observed that 71.2% of the IEs were due to MDROs and our experience (unpublished data) has shown that mortality in these patients is very high. However, with GTX we could salvage patients in 36 of the 47 IEs (76.6%); highlighting that GTX may especially be an important life-saving modality in neutropenic patients with MDR infections.

In general a lower response rate to GTX in fungal as compared to bacterial infections has been reported **[7,9,10,14,17,20,22,24,26–29,31–33]** even though a high susceptibility has also been reported **[19,25].** In a review article by Valentini *et al*, it is stated that higher doses of GTX were associated with improvement in fungal infections **[37].** However, in a study by Teofili *et al*, a dose dependent effect of GTX in fungal infections could not be demonstrated **[31].** Thus the efficacy and dose of GTX for fungal infections is still an area of investigation. In our series despite a high incidence of IEs due MDROs (71%), the resolution of the index infection and 30 day survival was high after GTX. One of the reasons could be that only 13% of IEs were due to fungal infections, highlighting the greater efficacy of GTX in bacterial infections, even though due to MDROs.

None of our patients demonstrated GTX related serious adverse events and GTX were well tolerated with mild TRALI in 6 of the 66 IE, which could be managed conservatively without requiring a ventilator support. In other studies also GTX have been demonstrated to be safe with a low incidence of side effects **[41].** Side effects mainly consist of pulmonary complications especially in patients with pre-existing pneumonia, and is rarely life threatening **[42].** There may be an exacerbation of pulmonary symptoms and radiological signs with high doses of GTX **[31].** We observed that the donors tolerated the apheresis procedure well except for mild bony pains in 6 donors, which required an analgesic drug.

Though a randomized controlled trial would have been ideal, but it is difficult to conduct ethically since in the event of a life-threatening infection especially when due to MDROs, GTX may be the only modality of treatment to salvage such patients and obviate mortality. For this reason most of the published studies are retrospective or prospective studies and there are very few randomized controlled trials (RCT) on the efficacy of GTX **[37].** In the recently concluded RCT RING study, a definite conclusion on the efficacy of GTX could not be arrived at because of a low accrual rate of the study, and a consequent decrease in the power to detect differences. One of the reasons for the low accrual rate was that several patients did not opt to participate in the trial or they opted out, as they did not like to be bound by the arm of the trial.

## Conclusion

In patients with severe neutropenic sepsis, where 71.2% of the IEs were due to MDROs, GTX at a median dose of 57.7 x10^9^ granulocytes/ IE or 10.4 x10^8^ granulocytes/Kg resulted in a resolution of the index infection in 68.2% IEs with a survival at 30 days of 67.7%. Administration of GTX within 7 days was associated with a significantly better survival. There was no difference in the 30 day survival between patients given GTX dose of ≥ 50x10^9^ per IE or those who were given GTX dose of < 50x10^9^ per IE. GTX were well tolerated and safe. To the best of our knowledge, this is the first study from India on large- scale use of GTX in both adult and pediatric patients with severe neutropenia. In a country like India, where there is an increasing emergence of bacterial infections due to MDROs, GTX may play a vital role especially when other antimicrobial therapies fail.
